# Association between the triglyceride glucose index and coronary collateralization in coronary artery disease patients with chronic total occlusion lesions

**DOI:** 10.1186/s12944-021-01574-x

**Published:** 2021-10-25

**Authors:** Ang Gao, Jinxing Liu, Chengping Hu, Yan Liu, Yong Zhu, Hongya Han, Yujie Zhou, Yingxin Zhao

**Affiliations:** 1 Department of cardiology, Beijing AnZhen Hospital, Capital Medical University, 100029 Beijing, China; 2 Beijing Institute of Heart Lung and Blood Vessel Disease, 100029 Beijing, China

**Keywords:** Triglyceride glucose index, Insulin resistance, Chronic total occlusion, Coronary collateralization

## Abstract

**Background:**

Recent studies have substantiated the role of the triglyceride glucose (TyG) index in predicting the prognosis of coronary artery disease (CAD) patients, while no relevant studies have revealed the association between the TyG index and coronary collateralization in the event of coronary chronic total occlusion (CTO). The current study intends to explore whether, or to what extent, the TyG index is associated with impaired collateralization in CAD patients with CTO lesions.

**Methods:**

The study enrolled 1093 CAD patients undergoing cardiac catheterization for at least one CTO lesion. Data were collected from the Beijing Anzhen Hospital record system. The degree of collaterals was determined according to the Rentrop classification system. The correlation between the TyG index and coronary collateralization was assessed.

**Results:**

Overall, 318 patients were included in a less developed collateralization (Rentrop classification 0-1) group. The TyG index was significantly higher in patients with impaired collateralization (9.3±0.65 vs. 8.8±0.53, *P*<0.001). After adjusting for various confounding factors, the TyG index remained correlated with the occurrence of impaired collateralization, with odds ratios (ORs) of 1.59 and 5.72 in the T2 and T3 group compared with the first tertile group (*P*<0.001). In addition, subgroup analysis showed that higher TyG index values remained strongly associated with increased risk of less developed collateralization. To compare the risk assessment efficacy for the formation of collateralization between the TyG index and other metabolic abnormality indicators, an area under the receiver-operating characteristic (ROC) curve (AUC) was obtained. A significant improvement in the risk assessment performance for impaired collateralization emerged when adding the TyG index into a baseline model.

**Conclusions:**

The increased TyG index is strongly associated with less developed collateralization in CAD patients with CTO lesions and its risk assessment performance is better than single metabolic abnormality indicators.

**Supplementary Information:**

The online version contains supplementary material available at 10.1186/s12944-021-01574-x.

## Introduction

CAD refers to the accumulative process of atherosclerotic plaques in the epicardial arteries and is a major cause of death worldwide. In recent years, the application of invasive strategies, such as percutaneous coronary intervention (PCI), coronary artery bypass grafting (CABG) and antithrombotic treatments, has greatly decreased the in-hospital major adverse cardiovascular events (MACEs) and mortality rates [1–3]. However, 5 %-10 % of patients continue to experience symptoms of angina pectoris despite optimal drug therapy and revascularization strategies, namely “no-option” patients [4]. The reasons that patients may not be suitable for revascularization include advanced age, multiple comorbidities and, most importantly, having complicated coronary lesions such as CTO. According to the recent data from the large clinical trials and the report from National Cardiovascular Data Registry [5–7], the success rate of the recanalization of CTO is significantly lower than that of non-CTO patients, and patients undergoing PCI for CTO require a higher contrast volume and longer fluoroscopy time, which exerts unfavourable effects on the prognosis of patients, especially for older patients and those with chronic kidney disease. Hence, invasive revascularization therapy may not be the optimal option for these patients. Coronary collateralization, as a network of arterio-arterial anastomotic connections between major epicardial arteries, could effectively alleviate the symptoms caused by cardiac ischaemia in the presence of severe obstructive CAD [8]. Studies have shown the protective role of well-developed coronary collateralization in improving the survival and prognosis of patients with CAD [9, 10]. While people with specific clinical characteristics tend to form less developed coronary collaterals [11, 12]. Previous studies found that there is an inverse association between coronary collateral growth and diabetes, metabolic syndrome (MetS), or its key parameter of insulin resistance (IR) [13]. The homeostasis model assessment-IR (HOMA-IR), an indicator of IR, is not very applicable for routine practice because insulin is not a common laboratory measurement for CAD patients, especially for those with nondiabetes. Hence, identifying a simple surrogate index of IR to evaluate the formation of coronary collateralization is of significance.

The TyG index, whose value is mainly determined by the fasting triglyceride and glucose levels, has been recognized as a novel surrogate marker of IR and has been proven to be strongly associated with HOMA-IR [14]. Evidence has shown that the TyG index can predict the presence of diabetes, prediabetes and MetS [15, 16]. Recent studies have focused on the association between the TyG index and cardiovascular complications and outcomes in those with CAD and show a significant correlation between the TyG index and coronary artery calcification [17], arterial stiffness [18] and MACEs in patients with CAD [19–22]. However, to date, there is no exclusive study that focused on the association between the TyG index and coronary collateralization in CAD patients with CTO lesions. Hence, this study aimed to investigate the association between the TyG index and coronary collateral grading and compare its risk diagnostic value with that of metabolic abnormality indicators.

## Methods

### Study population

**Fig. 1:**
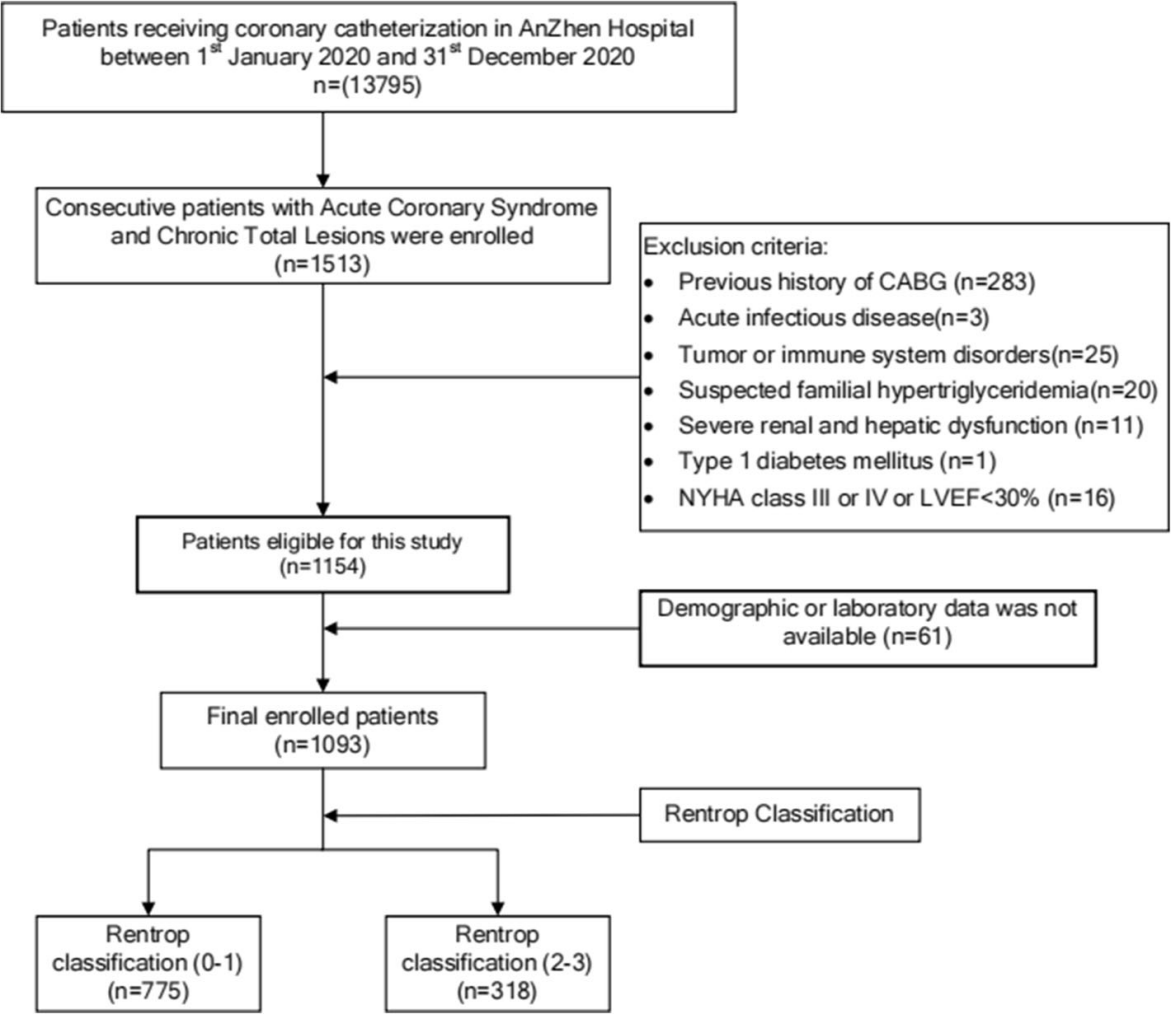
Flow chart of the study.

A total of 1093 patients who were diagnosed with CAD and with CTO lesion of at least one major epicardial coronary artery at Beijing AnZhen Hospital between 1st January 2020 and 31st December 2020 were recruited into this single-centre, observational study. CTO was defined as a coronary lesion with Thrombolysis In Myocardial Infarction flow grade 0 for at least 3 months [23]. The estimation of the duration of occlusion was based on the occurrence of myocardial infarction in the territory supplied by the occluded vessels, an abrupt worsening of existing angina or comparison with a prior angiogram. The detailed enrolment procedure is depicted in Fig. 1 and additional file 2.

### Disease definition

The diagnosis of type 2 diabetes mellitus (T2DM) was based on the previous diagnosis and treatment with glucose-lowering medication or recommendations from the American Diabetes Association, which includes (1) a fasting plasma glucose (FPG) level ≥126 mg/dL (7.0 mmol/L); (2) a 2-h postprandial glucose level≥200 mg/dL (11.1 mmol/L) during 75 g oral glucose tolerance test; (3) an glycated haemoglobin A1c (HbA1c) ≥6.5 %; and (4) classic symptoms of hyperglycaemia or hyperglycaemic crisis, with a random plasma glucose level ≥200 mg/dL (11.1 mmol/L) [24]. Hypertension was defined by the recommendations from the Europe Society of Cardiology/European Society of Hypertension: an office systolic blood pressure (SBP) value≥140 mmHg and/or a diastolic blood pressure (DBP) value≥90 mmHg or the use of antihypertensive agents in the past 2 weeks [25]. Dyslipidaemia was characterized by an increased total cholesterol (TC) or low-density lipoprotein cholesterol (LDL-C) and triglyceride (TG) levels or a decreased high-density lipoprotein cholesterol (HDL-C) level, according to the third report of the National Cholesterol Education Program [26]. MetS was defined according to the guidelines for T2DM by the Diabetes Branch of the Chinese Medical Association [27], using body mass index (BMI) for the replacement of waist circumference or obesity, that included three or more of the following abnormalities: (1) a high blood pressure ≥130/85 mmHg or treatment with antihypertensive drugs; (2) hypertriglyceridaemia (serum triglyceride level≥1.70 mmol/L or 150 mg/dL); (3) a low HDL-C level<1.04 mmol/L; (4) elevated fasting glucose level (≥6.1 mmol/L) or treatment with antidiabetic drugs; and (5) having overweight or obesity (BMI ≥25 kg/m²).

### Demographic and biochemical measurements

Demographic data were collected from the Beijing Anzhen Hospital medical information record system; left ventricular ejection fraction (LVEF) was calculated by echocardiography at admission; and blood samples were taken after overnight fasting (>8 h). Laboratory indicators were all calculated by standard techniques. The non-high-density lipoprotein cholesterol (non-HDL-C) was calculated as TC minus HDL-C. The TyG index was calculated as In (fasting glucose level [mg/dL]×fasting TG levels [mg/dL]/2). The TG-to-HDL-C ratio was calculated as TG/HDL-C. The study protocol was approved by the Institutional Review Board of The Anzhen Hospital, Beijing, China.

### Coronary angiography and collateral classification

The formation of coronary collateralization in CAD patients with CTO lesions was determined by cardiac catheterization, which was performed via radial or femoral access using 6 F diagnostic catheters. The angiogram results were independently evaluated and explained by two experienced interventional cardiologists who were blinded to this study. A Rentrop scoring system was used to assess the grading of coronary collateralization [28]: Grade 0, no visible filling of any collateral vessel; Grade 1, filling of the side branch via the collateral channel but without filling of the epicardial arteries; Grade 2, partial filling of the epicardial artery via collateral branches; and Grade 3, complete filling of the epicardial artery. In patients with more than 1 collateral branch, the highest grading was selected for the final analysis. Rentrop classification (0-1) was defined as less developed coronary collateralization. More vivid illustrations about the Rentrop classification system can be seen in Additional file 3 [28].

### Statistical analysis

As shown in Table 1, continuous variables are presented as the mean±standard deviation or median with interquartile range. Differences between the two groups were compared by Student’s t test or the Mann-Whitney U test for normally or nonnormally distributed continuous variates. Categorical variables are presented as frequencies and percentages, and difference between groups were performed by a chi-square test. Univariate logistic regression analysis was performed to preliminarily identify potential clinical risk indicators for less developed coronary collateralization, and a *P* value<0.05 was considered clinically significant. Pearson’s and Spearman’s correlation analyses were used to examine the association between the TyG index and the other risk indicators. To examine the independent performance of the TyG index for assessing the occurrence of impaired collateralization, participants were then divided into 3 groups according to TyG index tertiles (shown in Additional file 1). The differences between the groups were compared by one-way ANOVA or the Kruskal-Wallis H test for normally or nonnormally distributed variables. Differences in categorical variables between the groups were compared by the chi-square test or Fisher’s exact test. After adjusting for various potential risk indicators derived from univariate logistic regression analysis, a weighted multivariate logistic regression analysis was performed to calculate the ORs with 95 % confidence intervals (CIs) of the TyG index for the assessment of impaired collateralization. A subgroup analysis was further constructed to evaluate the association between the TyG index and less developed coronary collateralization in different subgroups. Finally, to investigate the incremental value of the TyG index on the discriminative performance beyond the baseline model, ROC curves were performed to compare the AUCs using De Long test. The incremental diagnostic value of different models for the less developed coronary collateralization was examined by net reclassification improvement (NRI) and integrated discrimination improvement (IDI). Statistical analysis was conducted using SPSS 23.0 (SPSS, Inc., Chicago, IL, USA) and R software (version 4.0.4) and MedCalc 19.1 (MedCalc software, Belgium). Two-sided *P* value < 0.05 was considered statistically significant.

**Table 1:** Baseline characteristics of enrolled population

	Total populations	Rentrop Classification (0-1)	Rentrop Classification (2-3)	*P*
	(*n*=1093)	(*n*=318)	(*n*=775)	
Demographic data				
Age (years)	58.76±10.17	58.0±10.11	59.1±10.19	0.064
Male sex, n (%)	936 (85.6 %)	270 (85 %)	666 (86 %)	0.659
BMI (Kg/m²)	26.4±3.30	26.7±3.21	26.2±3.32	0.019*
SBP (mmHg)	130.1±16.49	129.7±15.63	130.3±16.84	0.721
DBP (mmHg)	77.1±11.22	77.3±10.54	77.0±11.49	0.856
Current Smokers, n (%)	597 (55 %)	188 (59 %)	409 (53 %)	0.056
Medical history				
HTN, n (%)	734 (67 %)	206 (65 %)	528 (68 %)	0.284
T2DM, n (%)	463 (42 %)	147 (46 %)	316 (41 %)	0.098
MetS, n (%)				
Hypertriglyceridemia, n (%)	446 (41 %)	202 (64 %)	244 (32 %)	<0.001*
Hypercholesterolemia, n (%)	613 (56 %)	199 (63 %)	414 (53 %)	0.002*
Previous MI, n (%)	179 (16 %)	129 (17 %)	50 (16 %)	0.708
Previous Stroke, n (%)	109 (10 %)	39 (12 %)	70 (9 %)	0.105
Number of components of Mets				
0	36 (3 %)	6 (2 %)	30 (4 %)	0.095
1	142 (13 %)	26 (8 %)	116 (15 %)	0.002*
2	274 (25 %)	59 (19 %)	215 (28 %)	0.001*
3	296 (27 %)	95 (30 %)	201 (26 %)	0.183
4	240 (21 %)	85 (27 %)	155 (20 %)	0.015*
5	105 (10 %)	47 (15 %)	58 (7 %)	<0.001*
Laboratory measurement				
MONO, 10^12^/L	0.41±0.14	0.43±0.14	0.40±0.14	0.002*
eGFR	91.4±16.89	91.2±18.15	91.4±16.36	0.693
CREA, mmol/L	78.3±19.61	79.2±21.28	78.0±18.89	0.636
UREA, mmol/L	5.6±1.83	5.8±1.90	5.5±1.80	0.092
UA, mmol/L	355.4±94.97	369.8±97.57	349.6±93.32	<0.001
FBG, mmol/L	5.88 (5.11-7.25)	6.23 (5.22-8.10)	5.75 (5.06-7.00)	<0.001*
GA, %	14.4 (13.0-17.2)	14.5 (12.9-18.0)	14.4 (13.0-16.8)	0.455
HbA1C, %	6.1 (5.7-6.9)	6.2 (5.8-7.4)	6.1 (5.7-6.8)	0.001*
TyG index	9.3±0.65	9.3±0.65	8.8±0.53	<0.001
TC, mmol/L	3.9±0.99	4.1±1.07	3.8±0.94	<0.001
TG, mmol/L	1.7±0.91	2.2±1.11	1.6±0.73	<0.001
HDL-C, mmol/L	1.0±0.25	1.0±0.24	1.1±0.25	<0.001*
LDL-C, mmol/L	2.3±0.88	2.4±0.95	2.2±0.85	0.005*
Non-HDL-C, mmol/L	2.9±0.97	3.1±1.05	2.8±0.91	<0.001*
Hs CRP, mg/L	1.02 (0.52-2.64)	1.13 (0.68-3.16)	0.97 (0.48-2.38)	0.002*
LVEF, %	59.3±8.59	59.0±8.91	59.5±8.46	0.343
Cardiovascular Medication				
Antiplatelet therapy, n (%)	940 (86 %)	274 (86 %)	666 (86 %)	0.921
β-blockers, n (%)	660 (60 %)	200 (63 %)	460 (59 %)	0.277
ACEI/ARB, n (%)	360 (33 %)	112 (35 %)	248 (32 %)	0.304
Statins, n (%)	902 (83 %)	267 (84 %)	635 (82 %)	0.423
CCB, n (%)	305 (28 %)	80 (25 %)	225 (29 %)	0.195
Severity of CAD				0.912
One-vessel disease, n (%)	154 (14 %)	43 (14 %)	111 (14 %)	0.775
Two-vessel disease, n (%)	313 (29 %)	90 (28 %)	223 (29 %)	0.941
Three-vessel disease, n (%)	626 (57 %)	185 (58 %)	441 (57 %)	0.737
CTO related artery				0.078
RCA	528 (48 %)	139 (44 %)	389 (50 %)	0.054
LCX	192 (18 %)	55 (17 %)	137 (18 %)	0.93
LAD	373 (34 %)	124 (39 %)	249 (32 %)	0.035*
Grade of collaterals				
0	69 (6 %)	69 (22 %)	-	
1	249 (23 %)	249 (78 %)	-	
2	551 (50 %)	-	551 (71 %)	
3	224 (21 %)	-	224 (29 %)	
ISR-CTO	104 (10 %)	37 (12 %)	67 (9 %)	0.14

* Indicates the difference between two groups was statistically significant

Abbreviations: *BMI* Body mass index, *SBP* Systolic blood pressure, *DBP* Diastolic blood pressure, *HTN* Hypertension, *T2DM* Type2 diabetes mellitus, *PCI* Percutaneous coronary intervention, *MetS* Metabolic syndrome, *MI* Myocardial infarction, *MONO* Monocyte count, *eGFR* Estimated glomerular filtration rate, *CREA* Creatinine, *UREA* Urea, *UA* Uric acid, *FPG* Fasting plasma glucose, *GA* Glycated albumin, *HbA1c* Glycosylated hemoglobin A1c, *TyG* index Triglyceride glucose index, *TC* Total cholesterol, *TG* Triglyceride, *HDL-C* High-density lipoprotein cholesterol, *LDL-C* Low-density lipoprotein cholesterol, *non HDL-C* Non high-density lipoprotein cholesterol, *hs-CRP* High-sensitivity C-reactive protein, *LVEF* Left ventricular ejection fraction, *ACEI/ARB* Angiotensin-converting enzyme inhibitors / angiotensin receptor blockers, *CCB* Calcium channel blocker, *CAD* Coronary artery disease, *CTO* Chronic total lesion, *RCA* Right coronary artery, *LCX* Left circumflex coronary artery, *LAD* Left anterior descending artery, *ISR* In-stent restenosis

## Results

### Baseline characteristics

The demographic, laboratory and angiographic characteristics of the participants are shown in Table 1. They were divided into Rentrop classification (2-3) group (*n*=775) and Rentrop classification (0-1) group (*n*=318) according to the Rentrop scoring system. There was no difference in age or proportion of sex between the two groups, but most of the participants were males. Compared with those with well-developed collateralization, those with impaired one had higher BMIs and higher rates of hyperlipidaemia; Furthermore, although not very statistically significant, those in the Rentrop classification (0-1) group were more likely to be cigarette smokers and T2DM patients. The biochemical measurements showed that patients with less developed coronary collateralization have higher metabolic abnormality markers and inflammatory status. Coronary angiographic analysis indicated that the incidences of CTO in the right coronary artery (RCA), left circumflex artery (LCX) and left anterior artery (LAD) were 48 %, 18 % and 34 % respectively, with a difference of *P*=0.078 between two groups. The distribution of number of the diseased vessels shows no significantly different (*P*=0.912). It seems that RCA-related CTO lesions tended to be well developed (*P*=0.054), while CTO lesions located in LAD were likely to be less developed (*P*=0.035); Notably, the rate of impaired collateralization seemed to be higher in patients whose CTO lesions caused by In-Stent Restenosis (ISR) (*P*=0.140).

Table 2 showed that the TyG index is significantly associated with some cardiovascular risk factors. A positive correlation was found between the TyG index and Mets and its components, while a negative correlation was indicated with age, HDL-C levels and eGFRs.

**Table 2:** Correlation between the TyG index and recognized cardiovascular risk factors

	Correlation coefficient	*P*
Age	-0.067	0.027*
Gender	0.043	0.152
BMI	0.193	<0.001*
TC	0.305	<0.001*
LDL-C	0.182	<0.001*
HDL-C	-0.314	<0.001*
Non-HDL-C	0.393	<0.001*
HbA1C	0.387	<0.001*
GA	0.301	<0.001*
UA	0.176	<0.001*
eGFR	-0.073	0.015*
Hs CRP	0.08	0.008*
LVEF	0.012	0.710
components of Mets	0.624	<0.001*

* Indicates correlation between two factors was statistically significant

Abbreviations: *BMI* Body mass index, *TC* Total cholesterol, *LDL-C* Low-density lipoprotein cholesterol, *HDL-C* High-density lipoprotein cholesterol, *Non-HDL-C* Non-high-density lipoprotein cholesterol, *HbA1c* Glycosylated hemoglobin A1c, *GA* Glycated albumin, *eGFR* Estimated glomerular filtration rate, *UA* Uric acid, *HsCRP* High-sensitivity C-reactive protein, *LVEF* Left ventricular ejection fraction, *MetS* Metabolic syndrome

### Association between TyG index and coronary collateralization

**Fig. 2:**
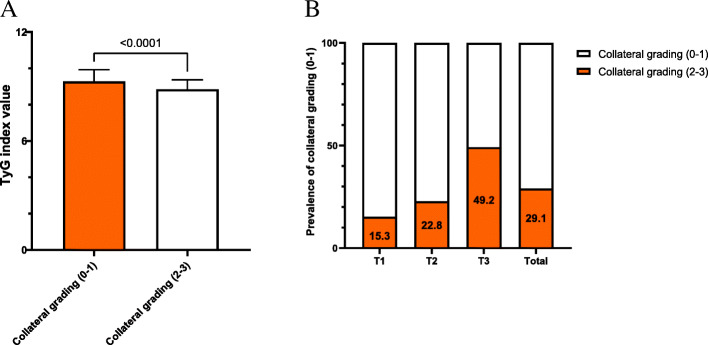
Comparison of the TyG index values between collateral grading (0-1) and (2-3) groups (**A**) and the prevalence of collateral grading (0-1) according to the TyG index tertiles (**B**). Abbreviation: TyG index: Triglyceride glucose index

**Fig. 3:**
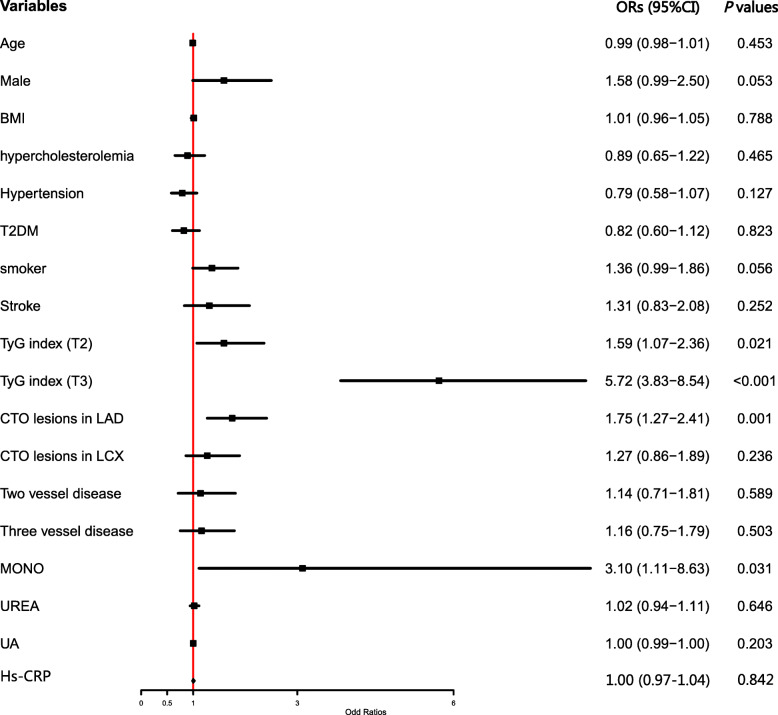
Forest plot of the multivariate logistic regression analysis model in patients with CTO lesions exploring the association between various risk factors and Rentrop classification (0-1). Abbreviations: CTO Chronic total occlusion, BMI Body mass index, T2DM Type2 diabetes mellitus, TyG index Triglyceride glucose index, MONO monocyte count, UREA: Urea, UA Uric acid, Hs CRP High-sensitivity C-reactive protein, RCA Right coronary artery, LCX Left circumflex artery, LAD Left anterior descending artery, CAD Coronary artery disease, ORs Odds ratios, CI Confidential interval

**Fig. 4:**
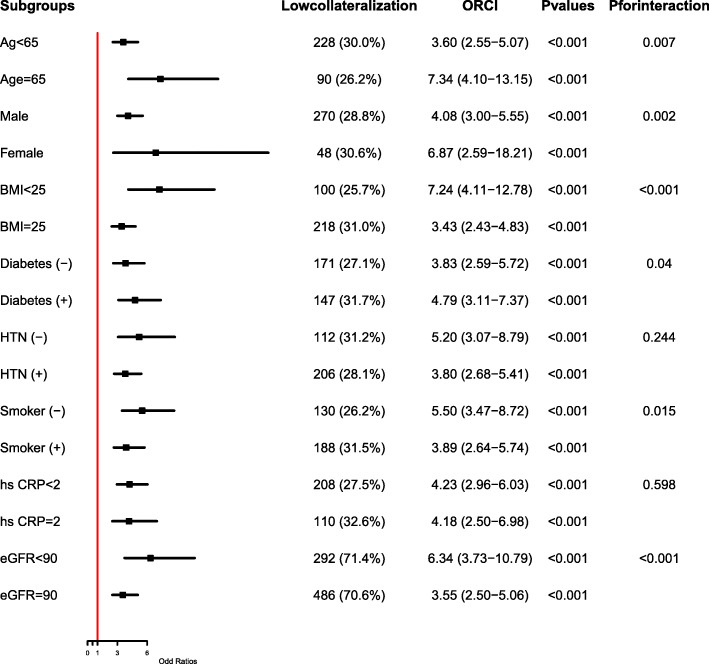
The impact of the TyG index on the prevalence of collateral grading (0-1) across subgroups of age, sex, BMI, glycometabolic status, hypertension, smoking status, inflammatory status and renal function. Abbreviations: BMI Body mass index, T2DM Type2 diabetes mellitus, HTN hypertension, Hs CRP high-sensitivity Creactive protein, eGFR Estimated glomerular filtration rate, ORs Odds ratios, CI confidence interval

**Fig. 5:**
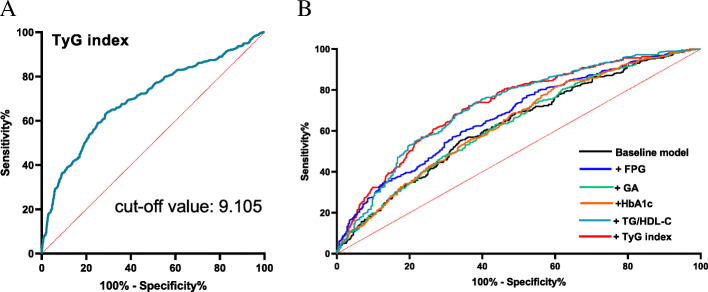
ROC curve of evaluating the diagnostic value of different models for Rentrop classification (0-1). (**A**) the ROC curve of TyG index for Rentrop classification (0-1); (**B**) the discriminative value of different models for evaluating collateral grading (0-1) using ROC curve. Abbreviations: TyG index Triglyceride glucose index, FPG Fasting plasma glucose, GA Glycated albumin, HbA1c glycosylated hemoglobin A1c, TG/HDL-C Triglyceride to High-density lipoprotein cholesterol ratio, ROC Receiver operating characteristics

As shown in Fig. 2 A, TyG index in patients with less developed collateralization was significantly higher than those with well-developed one (9.3±0.65 vs. 8.8±0.53, *P*<0.001). Once these participants were divided into 3 tertiles according to the TyG index, the proportion of poor collateralization increased stepwise from the lowest TyG index tertile to the highest one (15.3 % vs. 22.8 % vs. 49.2 %, *P*<0.001) (Fig. 2B). Multivariate logistic regression analysis was performed upon division, and Table 3 shows the ORs and 95 % CIs for impaired collateralization based on the TyG index tertiles. Unadjusted logistic regression analysis was performed to initially test the ORs of the TyG index for the less developed collateralization, and the ORs and 95 % CIs of the TyG index for the less developed collateralization in T2 and T3 group versus T1 group were 1.59 (1.07-2.36) and 5.72 (3.83-8.54) respectively after adjusting for various confounding factors. The factors influencing the formation of collateralization were listed in Fig. 3. Figure 4 presents the results of the subgroup analysis showing the relationship between TyG index and coronary collateralization in populations with different clinical characteristics. The adjusted ORs of TyG index for evaluating collateralization remained significant across all subgroups. Finally, Table 4 shows the incremental diagnostic value of the TyG index in the baseline model consisting of recognized risk factors, and finds its performance is better than other metabolic abnormality indicators. The improvement of the AUC for assessing the less developed collateralization was most significant when adding the TyG index to the baseline model with a best cut-off value of 9.105 (Fig. 5). Furthermore, the most significant enhancement in risk reclassification and discrimination was found after inclusion of the TyG index into baseline model, with a NRI of 0.238 (*P*<0.001) and an IDI of 0.103 (*P*<0.001) (Table 4).

**Table 3:** Association of the TyG index with coronary collateralization (Rentrop score 0-1) in multivariate logistic regression models

TyG index	Sample size of Rentrop classification (0-1)	ORs	95 % CI	*P*	*P* for trend
Unadjusted					<0.001
T1	56	Reference			
T2	83	1.63	1.12-2.37	0.011	
T3	179	5.34	3.76-7.58	<0.001	
Model 1					<0.001
T1	56	Reference			
T2	83	1.67	1.13-2.46	0.01	
T3	179	5.62	3.91-8.29	<0.001	
Model 2					<0.001
T1	56	Reference			
T2	83	1.59	1.08-2.35	0.02	
T3	179	5.31	3.58-7.87	<0.001	
Model 3					<0.001
T1	56	Reference			
T2	83	1.59	1.07-2.36	0.021	
T3	179	5.72	3.83-8.54	<0.001	

Model 1: adjust for age, sex, BMI, Smoking status, previous Stroke, Hypertension, Hypercholesterolemia, T2DM;

Model2: adjust for Model1+MONO+UREA+UA+hsCRP+LVEF;

Model3: adjust for Model2+severity of CAD+ISR-CTO+CTO related artery;

Abbreviations: *TyG* index Triglyceride glucose index, *ORs* Odds ratios, *CI* Confidence interval, *BMI* Body mass index, *T2DM* Type2 diabetes mellitus, *MONO* Monocyte count, *UREA* Urea, *UA* Uric acid, *Hs CRP* High-sensitivity C-reactive protein, *LVEF* Left ventricular ejection fraction, *CAD* Coronary artery disease, *ISR* In-stent restenosis, *CTO* Chronic total lesion

**Table 4:** Evaluate risk discriminative value of various models for coronary collateralization (Rentrop classification 0-1)

	ROC analysis			Categorical NRI			IDI		
	AUC	95 % CI	*P*	Estimation	95 % CI	*P*	Estimation	95 % CI	*P*
Baseline model	0.629	0.5932-0.6648		Reference			Reference		
๢FPG	0.673	0.6385-0.7075	0.001	0.067	0.0169-0.1171	0.009	0.0374	0.0236-0.0512	<0.001
๢GA	0.6323	0.5968-0.6678	0.523	0.005	-0.0523	0.699	0.0002	0.0001-0.0006	<0.098
๢HbA1c	0.638	0.6029-0.6732	0.186	0.001	-0.0568	0.991	0.0061	0.0012-0.0109	0.0137
๢TG/HDL-C	0.7217	0.6878-0.7556	<0.001	0.1592	0.0928-0.2255	<0.001	0.0878	0.0673-0.1083	<0.001
๢TyG index	0.7244	0.6917-0.7571	<0.001	0.238	0.1694-0.3066	<0.001	0.1036	0.0829-0.1244	<0.001

Abbreviations: *ROC* Receiver operating characteristic, *NRI* Net reclassification improvement, *IDI* Integrated discrimination improvement, *AUC* Area under ROC, *CI* Confidence interval, *FPG* Fasting plasma glucose, *GA* Glycated albumin, *HbA1c* Glycosylated hemoglobin A1c, *TG/HDL-C* Triglyceride to High-density lipoprotein cholesterol ratio, *TyG* index Triglyceride glucose index

## Discussion

The major findings of this study are as follows: (1) the TyG index was significantly higher in patients with impaired collateralizations than in those with good one; (2) the TyG index was independently associated with impaired collateralizations in CAD patients with CTO lesions; (3) the risk assessment value of TyG index for less developed collateralization was better than single metabolic abnormality indicators. Adding the TyG index to the baseline model shows the most significant incremental effect on risk discrimination for assessing the development of coronary collateral growth. To the best of our knowledge, this is the first study focusing on the correlation of the TyG index and coronary collateralization.

Coronary collaterals could expand four times larger, from a calibre of 10-200 μm to 100-800 μm, in the presence of CAD [29]. Well-developed coronary collaterals can exert a myocardial salvaging effect to limit the ischaemic area and preserve normal cardiac function [8]. Although the mechanism of coronary collateral growth (CCG) remained to be incompletely understood, clinical evidence has shown that CCG is impaired in patients with metabolic abnormality [13, 30]. The current study revealed that the TyG index, as a simple surrogate index of IR, is independently associated with less developed coronary collateralization, indicating that IR may play a pivotal role in the development of collateral circulation. IR, as a key parameter of MetS, affected factors that impaired the development of CCG, such as decreased expression of proangiogenic growth factors, increased production of reactive oxidative species (ROS) and continued endothelial dysfunction. The complete obstruction of the coronary artery leads to an elevated pressure gradient of arterioles and therefore increases tangential fluid shear stress to activate monocytes to release some angiogenic growth factors [31]. A recent study has shown that monocyte-derived dendritic cells tend to be functionally defective in the context of IR and thus increase vascular inflammation, which deteriorates the process of CCG [32]. IR-induced hyperglycaemia lead to overproduction of ROS by activating mitochondrial electron-transport chain, which is another reason for impaired collateralization [33]. IR is also characterized by the impairment of endothelial function, and IR is inversely associated with median colony forming unit endothelial cells, causing the decreased density of collaterals in response to cardiac ischaemia [13]. A recent study revealed that IR could decrease the expression of MicroRNA-21, an important mediator that regulates the secretion of nitric oxide (NO) and endothelin-1, thereby causing endothelial dysfunction [34].

The current study shows that the TyG index is strongly associated with the development of coronary collateralization after adjusting for various confounding factors. Apart from showing the independent association between the TyG index and poor collateralization, the current study also found that adding the TyG index to the baseline model had the strongest incremental effect on the risk assessment value for the formation of collateralization, thus highlighting the role of the TyG index in identifying CAD patients with extremely high cardiovascular risk. Several recent studies have compared the predictive value of the TyG index, FPG and HbA1c for cardiovascular events in CAD patients and attained similar results [35–37]. Discordance analysis conducted by Hu et al. [36] indicated that a high TyG index was always associated with a relatively high risk of cardiovascular events in CAD patients undergoing PCI when dividing those patients into different groups based on low/high FPG or HbA1c categories, highlighting its role of better predicting cardiovascular risks than FPG or HbA1c. Better predictive value for MACEs were also substantiated when adding TyG index into baseline risk model than FPG or HbA1c in acute myocardial infarction (AMI) patients with diabetes and non-ST-elevated acute coronary syndrome (NSTE-ACS) patients with non-diabetes [37]. Considering the relatively easier access to acquire the TyG index and its more accurately predictive value for cardiovascular events, present study provides evidence for using the TyG index in clinical practice to evaluate patients with high cardiovascular risks, especially for those without diabetes.

Given the high prevalence of MetS in CAD patients, there’s a need to develop convenient and low-cost screening tools to predict MetS and evaluate cardiovascular risks in those MetS-related CAD patients. Current study found the strongest link between MetS and TyG index (*r*=0.624, *P*<0.001). Recent studies have shown the efficacy of using TyG index to identify MetS. A population-based study found TyG index exhibited the best performance value in identifying MetS among obesity-related indices [38]. A large cross-sectional study from Korea also found that the value of the TyG index increased stepwise with the increasing components of MetS in middle-aged and older populations [39], which is in accordance with our current findings. In addition, TyG index could also predict the potential of developing MetS in healthy populations. A cohort study conducted by Lin et. al [15] showed that subjects with a higher TyG index were more likely to develop MetS in healthy populations over a 5-year follow-up. The current study provides clinical evidence for using the TyG index as an effective tool to evaluate MetS in CAD patients, suggesting the therapeutic potential of the TyG index in treating cardiometabolic diseases.

### Comparison with other studies and what does the current work add to the existing knowledge

Several previous observational studies have reported associations between metabolic abnormality markers with coronary collateralization [13, 40–42]. Kadi et al. found HDL-C was an independent risk factor for impaired coronary collateralization [41]. Another study exploring the role of TG to HDL-C ratio in discriminating the formation of coronary collateralization found that TG to HDL-C ratio was also an independent risk factor associated with impaired coronary collateralization in elderly patients in the context of AMI and coronary acute total occlusion [42]. However, the number of enrolled patients in these studies were relatively small and both studies failed to take the occluded time and coronary lesions into final analysis. The development of coronary collaterals is a time-dependent process and driven by shear force caused by the pressure gradient along the severely stenotic or occluded native vessels [43, 44]. To avoid the influence of these confounding factors on the final results, the present study investigated the association between metabolic indicators and coronary collateralization only in patients with totally occluded coronary vessels and the occluded time is over 3 months, namely CTO lesions. The growth of collateralization is also affected by inflammatory status. For example, Fan et al. [45] found that less developed coronary collateralization corresponded strongly with elevated C-reactive protein (CRP) levels. While no significant correlation was found between high-sensitivity CRP (hs-CRP) and coronary collateralization after adjustment in current study (Fig. 3). One plausible explanation behind the different results may be that the impact of inflammation is more or less mitigated by the strong association between TyG index and coronary collaterals. Further, other studies focusing on investigating the impact of glycometabolic status on coronary collateralization have found that glycometabolic indicators like FPG [13], glycated albumin (GA) [40] are all reliable predictors for the development of coronary collaterals in patients with CTO lesions but these studies did not compare the discriminative value for evaluating the formation of coronary collaterals between different models. The current study explore the association between TyG index and coronary collaterals in a relatively large number of patients with CTO lesions and found TyG index showed the strongest discriminative value for the development of coronary collaterals (Fig. 5; Table 4), indicating IR may play a pivotal role in the mechanisms inducing impaired coronary collateralizations and thus providing new therapeutic and risk management target in advanced CAD patients unsuitable for revascularization therapies.

### Study strengths and limitations

This single-center observational study investigated the correlation between the value of TyG index and the extent of coronary collateral circulation in patients with CTO lesions, which emphasizes the importance of mediating IR or metabolic abnormality in those patients with advanced CAD. The major strength of the present study was the relatively large number of enrolled subjects. Additionally, TyG index showed the highest discriminative value of poor coronary collateralization among metabolic indicators, which offered a novel therapeutic target for those advanced CAD patients not suitable for coronary revascularization. Meanwhile, several limitations should be acknowledged: (1) The single-centre design may limit the generalizability of the results, but the characteristics of the study population are similar to those from other large multicentre studies [46], which somehow supports the validity and applicability of the results in other cohorts. (2) The nature of study design is only suitable for detecting the association but incapable of assessing the causality between TyG index and coronary collateralization. (3) Although the effects of the established risk factors were statistically adjusted in multivariate logistic regression models, the adjustment cannot entirely remove confounding factors like congenital artery branching, inflammatory cytokines, angiographic growth factors, etc. (4) More detailed information about the TG-lowering drugs like fibrates and other agents, which would influence the level of TyG index, is relatively scarce. (5) The evaluation of coronary collateralization in current study was based on Rentrop scoring system for its easy use in clinical practice, but collateral collaterals should be more accurately assessed by collateral flow index. (6) Finally, HOMA-IR was not analyzed and compared with TyG index because HOMA-IR is not common laboratory indicator evaluating CAD patients, especially for those without diabetes. Despite these limitations, this study has potential implications for practice because it is the first to investigate the association between TyG index and coronary collateralization.

## Conclusions

In conclusion, TyG index is independently associated with the development of coronary collaterals and its discriminative value are better than single metabolic abnormality indicators. The findings may provide a better therapeutic target to manage advanced CAD patients, especially for those without diabetes. In addition, it is also necessary to evaluate the role of therapies targeting at IR in improving prognosis of these patients.

## Supplementary information

Additional file 1 Baseline characteristics of patients stratified by tertile of the TyG index

**Additional file 2**

**Additional file 3**

## Data Availability

The datasets and materials mentioned above are available from the authors.

## Abbreviations

TyG: index Triglyceride glucose index
ACS: Acute coronary syndrome
NSTE-ACS: NonST-segment elevation acute coronary syndrome
AMI: Acute myocardial infarction
CTO: Chronic total occlusion
PCI: Percutaneous coronary intervention
CABG: Coronary artery bypass grafting
HbA1c: Glycosylated haemoglobin A1c
IR: Insulin resistance
ROC: Receiver-operating characteristic
AUC: Area under curve
NRI: Net reclassification improvement
IDI: Integrated discrimination improvement
MACEs: Major adverse cardiovascular events
ISR: In-Stent Restenosis
MetS: Metabolic syndrome
HOMA-IR: Homeostasis model assessment of insulin resistance
T2DM: Type 2 diabetes mellitus
eGFR: Estimated glomerular filtration rate
SBP: Systolic blood pressure
DBP: Diastolic blood pressure
TC: Total cholesterol,
LDL-C: Low-density lipoprotein cholesterol
TG: Triglycerides
HDL-C: High-density lipoprotein cholesterol
non-HDL-C: Non-highdensity lipoprotein cholesterol
BMI: Body mass index
LVEF: Left ventricular ejection fraction
FPG: Fasting plasma glucose
GA: Glycosylated albumin
hs-CRP: High-sensitivity C-reactive protein
RCA: Right coronary artery
LCX: Left circumflex artery
LAD: Left anterior descending artery
CCG: Coronary collateral growth
NO: nitric oxide
ROS: Reactive oxidative species
