# Supplementary material for: Association between the triglyceride glucose index and coronary collateralization in coronary artery disease patients with chronic total occlusion lesions
Source: Lipids Health Dis. 2021 Oct 25;20:140. doi: 10.1186/s12944-021-01574-x (PMC8543811; doi:10.1186/s12944-021-01574-x)
Supplement: Supplementary file 1 — Baseline characteristics of patients stratified by tertile of the TyG index [file 12944_2021_1574_MOESM1_ESM.docx]

**Additional file1** Baseline characteristics of patients stratified by tertile of the TyG index

| TyG index | Total | T1 (n=365) | T2 (n=364) | T3 (n=364) |  | *P* |
| --- | --- | --- | --- | --- | --- | --- |
|  |  | （T1≤8.66） | （8.67＜T2＜9.18） | (T3≥9.19) |  |  |
| Demographic data |  |  |  |  |  |  |
| Age (years) | 58.76±10.17 | 59.90±9.95 | 58.69±10.05 | 57.69±10.41 |  | 0.013* |
| Male sex, n (%) | 936 (85.6%) | 320 (87.7%) | 306 (84.1%) | 310 (85.2%) |  | 0.363 |
| BMI (Kg/m²) | 26.4±3.30 | 25.35±3.00 | 26.76±3.45 | 26.98±3.19 |  | <0.001* |
| SBP (mmHg) | 130.1±16.49 | 129.80±16.45 | 130.53±16.57 | 130.12±16.49 |  | 0.838 |
| DBP (mmHg) | 77.1±11.22 | 76.67±11.01 | 77.19±11.18 | 77.44±11.48 |  | 0.643 |
| Current Smokers, n (%) | 597 (54.6%) | 194 (53.2%) | 196 (53.8%) | 207 (56.9%) |  | 0.563 |
| Medical history |  |  |  |  |  |  |
| Hypertension, n (%) | 734 (67.2%) | 236 (64.7%) | 252 (69.2%) | 246 (67.6%) |  | 0.412 |
| T2DM, n (%) | 463 (42.4%) | 90 (24.7%) | 155 (42.6%) | 218 (59.9%) |  | <0.001* |
| MetS, n (%) |  |  |  |  |  |  |
| Hypercholesterolemia, n (%) | 613 (56.1%) | 143 (39.2%) | 201 (55.2%) | 269 (73.9%) |  | <0.001* |
| Hypertriglyceridemia, n (%) | 446 (40.8%) | 2 (0.5%) | 135 (37.1%) | 309 (84.9%) |  | <0.001* |
| Previous Stroke, n (%) | 109 (10.0%) | 30 (8.2%) | 33 (9.1%) | 46 (12.6%) |  | 0.107 |
| Laboratory measurement |  |  |  |  |  |  |
| MONO, 10^12/L | 0.41±0.14 | 0.39±0.14 | 0.41±0.13 | 0.42±0.15 |  | 0.021* |
| eGFR | 91.4±16.89 | 93.17±14.01 | 90.21±17.55 | 90.97±18.24 |  | 0.046* |
| CREA, mmol/L | 78.3±19.61 | 75.50±15.85 | 79.65±21.01 | 79.88±21.25 |  | 0.003* |
| UREA, mmol/L | 5.6±1.83 | 5.39±1.63 | 5.54±1.83 | 5.83±1.99 |  | 0.005* |
| UA, mmol/L | 355.4±94.97 | 334.39±82.60 | 360.38±94.54 | 371.91±103.07 |  | <0.001* |
| FPG, mmol/L | 5.88 (5.11-7.25) | 5.24 (4.78-5.80) | 5.87 (5.18-6.80) | 7.50 (5.91-9.29) |  | <0.001* |
| GA, % | 14.39 (12.95-17.19) | 13.78 (12.75-15.49) | 14.29 (12.91-16.76) | 15.68 (13.37-19.56) |  | <0.001* |
| HbA1C, % | 6.10 (5.70-6.90) | 5.80 (5.60-6.20) | 6.15 (5.70-6.80) | 6.60 (6.00-7.90) |  | <0.001* |
| TC, mmol/L | 3.9±0.99 | 3.61±0.87 | 3.87±0.97 | 4.28±1.02 |  | <0.001* |
| TG, mmol/L | 1.7±0.91 | 1.02±0.25 | 1.60±0.37 | 2.66±0.94 |  | <0.001* |
| HDL-C, mmol/L | 1.0±0.25 | 1.13±0.25 | 1.04±0.25 | 0.95±0.20 |  | <0.001* |
| LDL-C, mmol/L | 2.3±0.88 | 2.08±0.79 | 2.26±0.88 | 2.46±0.93 |  | <0.001* |
| Non-HDL-C, mmol/L | 2.9±0.97 | 2.48±0.81 | 2.82±0.91 | 3.33±0.97 |  | <0.001* |
| Hs CRP, mg/L | 1.02 (0.52-2.64) | 0.80 (0.42-2.03) | 1.10 (0.57-2.69) | 1.15 (0.68-3.08) |  | <0.001* |
| LVEF, % | 59.3±8.59 | 59.40±7.86 | 59.17±8.17 | 59.32±8.33 |  | 0.927 |
| Angiography data |  |  |  |  |  |  |
| Severity of CAD |  |  |  |  |  | 0.494 |
| One-vessel disease, n (%) | 154 (14%) | 57 (15.6%) | 53 (14.6%) | 44 (12.1%) |  | 0.373 |
| Two-vessel disease, n (%) | 313 (29%) | 102 (27.9%) | 111 (30.5%) | 100 (27.5%) |  | 0.625 |
| Three-vessel disease, n (%) | 626 (57%) | 220 (60.3%) | 200 (54.9%) | 220 (60.4%) |  | 0.301 |
| CTO related artery |  |  |  |  |  | 0.044* |
| RCA, n (%) | 528 (48.3%) | 169 (46.3%) | 160 (44.0%) | 199 (54.7%) |  | 0.010* |
| LCX, n (%) | 192 (17.6%) | 65 (17.8%) | 67 (18.4%) | 60 (16.5%) |  | 0.784 |
| LAD, n (%) | 373 (34.1%) | 131 (35.9%) | 137 (37.6%) | 105 (28.8%) |  | 0.030* |
| ISR-CTO, n (%) | 104 (9.5%) | 36 (9.9%) | 34 (9.3%) | 34 (9.3%) |  | 0.962 |

* indicates difference between groups were statistically significant

Abbreviations: BMI Body mass index, SBP Systolic blood pressure, DBP Diastolic blood pressure, T2DM Type2 diabetes mellitus, PCI Percutaneous coronary intervention, MetS Metabolic syndrome, MONO Monocyte count, eGFR Estimated glomerular filtration rate, CREA Creatinine, UREA Urea, UA Uric acid, FPG Fasting plasma glucose, GA Glycated albumin, HbA1c Glycosylated hemoglobin A1c, TyG index Triglyceride glucose index, TC Total cholesterol, TG Triglyceride, HDL-C High-density lipoprotein cholesterol, LDL-C Low-density lipoprotein cholesterol, Non HDL-C Non high-density lipoprotein cholesterol, Hs CRP High-sensitivity C-reactive protein, LVEF Left ventricular ejection fraction, ACEI/ARB Angiotensin-converting enzyme inhibitors / Angiotensin receptor blockers, CCB Calcium channel blocker, CAD Coronary artery disease, CTO Chronic total lesion, RCA Right coronary artery, LCX Left circumflex coronary artery, LAD Left anterior descending artery, ISR In-stent restenosis
