# Supplementary material for: Association between the triglyceride glucose index and coronary collateralization in coronary artery disease patients with chronic total occlusion lesions
Source: Lipids Health Dis. 2021 Oct 25;20:140. doi: 10.1186/s12944-021-01574-x (PMC8543811; doi:10.1186/s12944-021-01574-x)
Supplement: Supplementary file 2 — Additional file 2 [file 12944_2021_1574_MOESM2_ESM.docx]

**Additional file 2**

| Inclusion criteria | Exclusion criteria |
| --- | --- |
| 1) Age ≥ 18 | 1) previous history of CABG (n=283) |
| 2) Patients diagnosed with CAD | 2) acute infectious disease (n=3) |
| 3) CAD patients whose coronary vessels are totally occluded for over 3 months | 3) malignant tumor or immune system disease (n=25) |
|  | 4) suspected familial hypertriglyceridemia (n=20) |
|  | 5) severe renal and hepatic dysfunction (n=11) |
|  | 6) T1DM (n=1) |
|  | 7) NYHA class III-IV or LVEF<30% (n=16) |
|  | 8) baseline demographic data are not available (n=61) |

Abbreviations CAD Coronary artery disease, CABG Coronary artery bridge grafting, T1DM Type 1 diabetes mellitus, NYHA New York Heart Association
