# Supplementary material for: Association between the triglyceride glucose index and coronary collateralization in coronary artery disease patients with chronic total occlusion lesions
Source: Lipids Health Dis. 2021 Oct 25;20:140. doi: 10.1186/s12944-021-01574-x (PMC8543811; doi:10.1186/s12944-021-01574-x)
Supplement: Supplementary file 3 — Additional file 3 [file 12944_2021_1574_MOESM3_ESM.docx]

**Additional file 3**


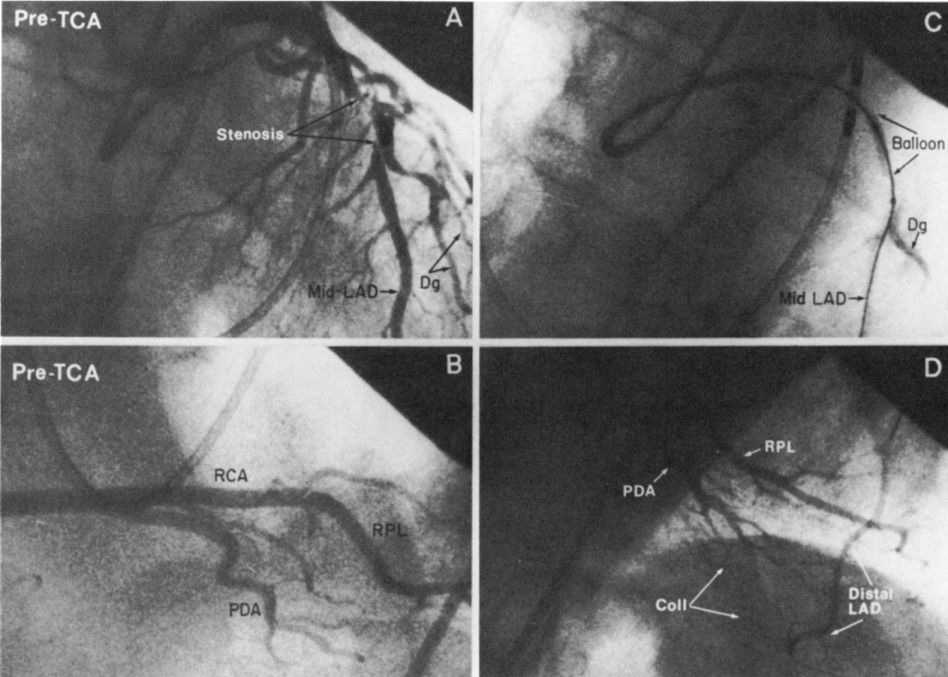


Illustrations about the Rentrop classification system by Rentrop et. al[1].

Fig. A showed there is a 95% stenosis in the left anterior descending artery

Fig. B showed no visible collateral branch can be seen Fig.

C and D showed Rentrop classification 3 can be gradually seen during balloon inflation

1. Rentrop KP, Cohen M, Blanke H, Phillips RA: **Changes in collateral channel filling immediately after controlled coronary artery occlusion by an angioplasty balloon in human subjects.** *J Am Coll Cardiol* 1985, **5:**587-592.
